# Polyampholytic Hydrogels from Chitosan Macromonomers with Aryl-Mono and Di-Sulfonated Groups: An Approach to the Removal of Copper Ions and Ciprofloxacin in Aqueous Solutions

**DOI:** 10.3390/gels11080622

**Published:** 2025-08-08

**Authors:** Diana Montoya-Rodríguez, Alexis Salas, Manuel F. Meléndrez, Elizabeth R. Gillies, Daniel A. Palacio

**Affiliations:** 1 Departamento de Polímeros, Facultad de Ciencias Químicas, Universidad de Concepción, Edmundo Larenas 129, Concepcion 4070371, Chile; dianamontoya@udec.cl; 2 Department of Mechanical Engineering (DIM), Faculty of Engineering, University of Concepción, 219 Edmundo Larenas, Concepcion 4070409, Chile; alesalas@udec.cl; 3 Facultad de Ciencias para el Cuidado de la Salud, Universidad San Sebastián, Campus Las Tres Pascuales, Lientur 1457, Concepcion 4060000, Chile; manuel.melendrez@uss.cl; 4 Department of Chemistry, The University of Western Ontario, London, ON N6A 5B7, Canada; egillie@uwo.ca; 5 Department of Chemical and Biochemical Engineering, The University of Western Ontario, London, ON N6A 5B7, Canada

**Keywords:** chitosan, sulfonation, polyampholytic hydrogels, copper removal, ciprofloxacin removal

## Abstract

Functional hydrogels have significant potential for applications in the pharmaceutical, agricultural, and environmental sectors. This study focuses on the synthesis of polyampholytic hydrogels through free radical polymerization using functionalized chitosans. The chitosan was modified with mono and disulfonic groups at different temperatures (25 °C and 60 °C) and reaction times (1, 8, 24 h), followed by further modification with glycidyl methacrylate to introduce vinyl groups into the polymers structure. The modified polymers were analyzed using proton nuclear magnetic resonance, Fourier transform infrared, scanning electron spectroscopy, thermogravimetric analysis, and solubility tests. Specifically, 0.74 mmol/g and 1.58 mmol/g of the primary amine groups available in the chitosan chain (out of a total of 4.93 mmol/g) were substituted with mono- and disulfonic groups, respectively. Following treatment with glycidyl methacrylate, 3.39 mmol/g and 2.21 mmol/g of the remaining primary amine groups in the mono- and disulfonic polymers, respectively, were substituted. The hydrogels obtained by the modified polymers at optimal conditions of 1 h and 25 °C, were characterized by the techniques already mentioned in addition to rheological tests, and water absorption studies across different pHs. The hydrogels demonstrated potential for environmental remediation, particularly in adsorptions of ciprofloxacin (CPX) and copper (Cu^2+^) from aqueous solutions at pH 7, achieving adsorption efficiencies of 24–25% for CPX and 83% for Cu^2+^. The results suggest that the synthesized hydrogels could provide an eco-friendly and efficient solution to challenges in wastewater treatment.

## 1. Introduction

Hydrogels (HGs) are three-dimensional structures composed of high molecular weight hydrophilic polymers that are either physically or chemically cross-linked, providing them with a high capacity for water absorption. This property makes them desirable for applications across various fields, including environmental remediation, tissue engineering, regenerative medicine, drug administration, and absorbent materials, among others [1,2]. In particular, hydrogels with polyampholytic properties have aroused special interest in environmental remediation. This is because polyampholytic HGs combine intrinsic characteristics of this type of materials such as porosity, with the ability to participate in interactions such as hydrogen bonding, van der Waals and electrostatic forces, cation exchange and complex formation, which improves their potential for the adsorption of pollutants [3]. Among the polymers used to obtain this type of hydrogels, the following stand out: polyacrylamide, cyclodextrin, chitosan, polyacrylate, and cellulose, because their combination has polyampholytic properties conferred by the combination of anionic and cationic polymers. Others are obtained from the functionalization of these polymers with compounds such as 1,3-diaminopropane, 1,4-diaminobutane, graphene oxide, among others [4,5,6,7,8,9].

Chitin stands out as the second most abundant natural polymer and one of the high molecular weight polymers used to obtain functional HGs. It has a linear structure composed of poly[β-(1-4)-2-acetamide-2-deoxy-D-glucopyranose] [10]. Through the partial deacetylation of chitin, chitosan is obtained, a linear cationic polysaccharide consisting of β-(1-4)-2-deoxy-2-amino-D-glucopyranose (D-glucosamine) and β-(1-4)-2-deoxy-2-acetamido-D-glucopyranose (N-acetyl-D-glucosamine) units [11]. Chitosan has been widely studied in various fields due to its unique properties, including biodegradability, hemocompatibility, biocompatibility, and antimicrobial activity, which make it suitable for numerous biological, chemical, and medical applications. The distinctive structure of chitosan, containing reactive amino and hydroxyl groups, allows for extensive chemical modification that enhances its solubility and reactivity in different environments [12,13].

Extensive research has been conducted to develop chemical, physical or enzymatic modifications to chitosan to enhance or introduce new properties that can be applied across diverse fields, including food, pharmaceutical, biotechnological, medical, textile, paper, agricultural, and environmental applications [11,14,15]. Several chemical modification strategies have been explored, depending on the desired applications and properties to be improved. For instance, modifications such as alkylation, acylation, hydroxyalkylation, carboxyalkylation, phosphorylation, azidation, and thiolation have been explored [16,17,18].

The research described here used reductive amination to enhance the absorption of different contaminants through chitosan functionalization. Specifically, our aim was to modify chitosan with sulfonic acid groups to provide anionic charge, supported by previous studies showing the effectiveness of these functional groups in the adsorption of pollutants [19,20,21]. The objective was to improve the solubility of chitosan at basic pH and allow the formation of polyampholytic HGs with characteristics that favor their adsorbent properties and interactions with contaminants. Although the sulfonation approach has been widely explored for biomedical applications and the development of ion exchange filters, most research has focused on the synthesis and characterization of these chitosan derivatives, with less emphasis on their use. Therefore, in this research we propose to go beyond what has already been reported in the literature on the chitosan functionalization, obtaining hydrogels with polyampholytic characteristics conferred by the incorporation of anionic functional groups, for the removal of contaminants in aqueous matrices [19,21,22].

The hypothesis of the present work is that the modification of chitosan with anionic groups, to obtain polyampholytic hydrogels, favors its ability to absorb different types of contaminants in aqueous media. This strategy represents a significant advance by combining the versatility of sulfonic groups with the water retention properties and biocompatibility of chitosan, thus offering an innovative and effective solution to environmental challenges related to water purification. This is particularly relevant in Chile, a country that faces increasing pressure on its freshwater resources due to mining, agriculture, and population growth, all of which contribute to the presence of persistent pollutants in surface and groundwater systems. In order to explore the first application of the polyampholytic hydrogel, two representative contaminants of concern in Chile were selected, Cu^2+^ and ciprofloxacin (CPX), whose cationic and ampholytic nature, respectively, could favor their electrostatic and hydrogen bonding interaction with the hydrogel. CPX is a broad-spectrum antibiotic, which is used in both humans and animals, ranking among the best-selling therapeutics in Chile [23]. Its metabolism is incomplete, so generally a large percentage of the dose is excreted through the urine and feces [24,25], thereby contaminating aquatic ecosystems. This contamination can result in the generation of resistant bacteria, insertion into the food chain and adverse effects such as an impact on the trophic abundance of some species after only one month of exposure [26,27,28].

Regarding metal ions, Chile is one of the countries with the highest copper production worldwide, with the potential for copper ion accumulation in the environment and its contamination of the food chain. Copper can bioaccumulate, leading to negative health effects such as an increased rates of cancer [29,30,31]. Consequently, researchers have designed various HG-type materials based on polymers such as lignin, cellulose, graphene or chitosan to adsorb copper ions [32,33,34,35,36,37]. In Chile, copper-related pollution is especially critical in regions such as Antofagasta and Atacama, where mining wastewater has led to elevated concentrations of heavy metals in rivers and aquifers, posing significant risks to both environmental and human health [38]. The development of hydrogels capable of selectively capturing such metals from contaminated waters aligns with the country’s need for accessible and efficient water treatment technologies.

To evaluate the hypothesis, this work begins by describing the preparation of sulfonated polymers through reductive amination, applying different reaction times and temperatures ranging between 1 and 24 h and 25 to 60 °C, to choose those with higher degrees of functionalization. The best conditions were found to be one hour of reaction at 25 °C, reaching a DS of 15 and 32%. The resulting polymers were then modified with glycidyl methacrylate (GMA), to incorporate vinyl bonds that would participate in the subsequent free radical polymerization to form the hydrogels. Free radical polymerization is a relatively simple, versatile, and low-cost crosslinking method [39]. These polyampholytic hydrogels were characterized by FTIR, TGA, and SEM, demonstrating 20 to 21 times the initial mass increase in swelling processes. Finally, the introduction of sulfonic groups into chitosan significantly enhances its water solubility and swelling properties, which makes it particularly suitable for environmental applications such as heavy metal and pharmaceutical removal from water. Our findings suggest that these materials have the potential to address challenges in wastewater treatment, offering an eco-friendly and efficient solution.

## 2. Results and Discussion

### 2.1. Functionalization of CTS with Sodium Sulfonates

#### 2.1.1. Characterizations by FTIR, ^1^H-NMR, and TGA

**Figure 1:**
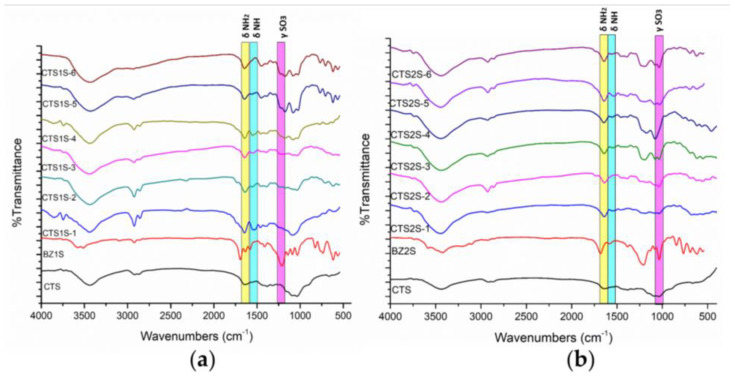
FTIR spectra for CTS and CTS modified with sulfonic groups. (**a**) Polymers obtained by modifying CTS with FB1S. (**b**) Polymers obtained by modifying CTS with FB2S.

**Figure 2:**
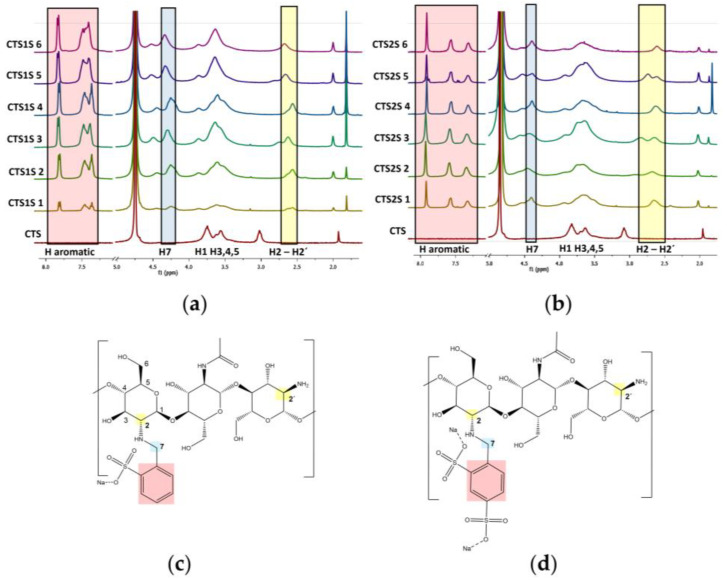
^1^H-NMR spectra of polymers modified with sulfonic groups. (**a**) Polymers obtained by modifying chitosan with FB1S. (**b**) Polymers obtained by modifying chitosan with FB2S. (**c**) Expected structures of the polymers modified with FB1S. (**d**) Expected structures of the polymers modified with FB2S.

The chitosan derivates modified with 2-formylbenzylsulfonic (FB1S) and 4-formyl-1,3-benzenedisulfonic (FB2S) under different time and temperature conditions were initially characterized by FTIR spectroscopy to determine whether the evaluated parameters, such as temperature and reaction time, led to the effective functionalization of the biopolymer. The FTIR spectra are shown in Figure 1 following the nomenclature proposed in Table 2, according to the modification conditions. The results reveal two characteristic bands for both FB1S and FB2S, indicating the successful functionalization of the polymer. The first notable signal was observed at 1450 cm^−1^, corresponding to the bending vibrations of the substituted amine groups (Figure 2c,d). Additionally, a series of peaks attributed to the stretching of the S=O bond from the sulfonic groups appeared between 1170 and 1190 cm^−1^ in the spectra of the functionalized derivatives [40,41,42].

^1^H-NMR spectra of the biopolymers are shown in Figure 2, where the analysis of two regions of the spectra supports the functionalization of the CTS. First, new peaks appeared between 7.1 and 7.5 ppm corresponding to the protons on the aromatic group. In addition, functionalization resulted in peaks between 4.2 and 4.5 ppm, corresponding to the benzylic protons [43,44]. These results qualitatively indicated that all the conditions evaluated led to a modification of the CTS. To quantify the degree of substitution (DS), Equation (1) was used where the integrals of the peaks corresponding to the aromatic protons (Ar), the proton from the alpha ring adjacent to the secondary amine (H_2_) or primary amine (H_2_^′^), as well as the degree of deacetylation of the starting chitosan (DDA) and the number of aromatic protons, being 3 and 4 for FB1S and FB2S, respectively, were considered [44]. The results obtained are presented in Table 1. It was determined that the conditions that lead to higher degrees of modification were one hour of reaction at 25 °C, reaching a DS of 15 and 32% for CTS1S and CTS2S, respectively. Thus, CTS1S remains structurally with 4.19 mmol of residual primary amino groups, while CTS2S—with 3.35 mmol/g of primary amine groups.

%DS = (Ar/n)/((H_2_ + H_2_′)/DDA) × 100 (1)

The degrees of substitution determined by ^1^H-NMR spectroscopy were complemented by energy-dispersive X-ray scanning electron microscopy (SEM-EDS) analyses. Specifically, the percentages of the elements of interest such as carbon, nitrogen, oxygen, and sulfur were quantified (Table 1), where the average results of random points in the samples of the polymers are presented. Sulfur was of special interest, as a quantitative indicator of the degree of functionalization. The heterogeneity of the sample makes it difficult to establish comparisons between the results of both techniques, since the modification with sulfonic groups depends on the distribution and availability of the amino groups. However, it was observed that the polymers with higher percentages of sulfur (%S) were prepared with a reaction time of 1 h at 25 °C.

**Figure 3:**
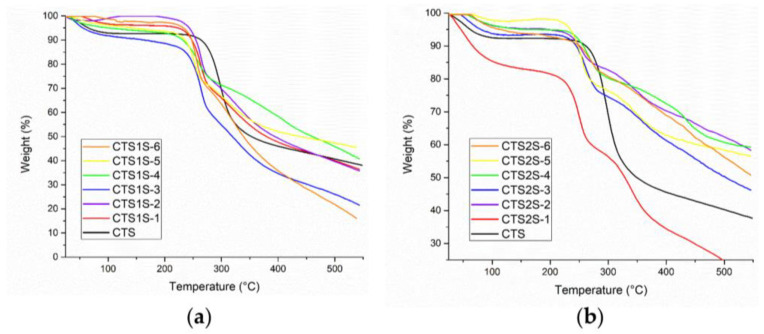
Results obtained by thermogravimetric analysis of the modified polymers at different time and temperature conditions. (**a**) CTS1S polymers. (**b**) CTS2S polymers.

In TGA (See Figure 3), the curves obtained allowed the identification of the stages in which the polymers degraded, where the gradients in weight percentage represented abrupt changes in weight loss over specific temperature intervals. Figure 2 shows the results obtained for the modified polymers, in comparison with the commercial CTS sample. For CTS, the decomposition was evident in two steps—the first, with a maximum rate at 54 °C, corresponding to the loss of water, and the second at 297 °C associated with the decomposition and carbonization of the sugar ring of the CTS. On the other hand, all modified CTS samples decomposed in three steps.

The first step, corresponding to the loss of water was observed at temperatures less than 70 °C. The second step had a maximum rate of mass loss between 260 and 290 °C, and was attributed to the decomposition of the CTS sugar ring as well as the amine and sulfonic functional groups. Finally, the decomposition step corresponding to the organic residues of chitosan and the aryl groups occurred between 340 and 440 °C [12,17]. The results obtained show a thermal stability associated with the electrostatic interactions produced by the modification of sulfonic groups [12,17].

Table 1 shows the percentages of weight loss obtained from the analysis of the TGA graphs, corresponding to the third step, attributed to the modification by the aryl-sulfonated groups for each of the samples, with the objective of contrasting the weight losses for products obtained using different reaction conditions. In both cases, the greatest weight losses associated with the aryl groups were obtained for polymers prepared using the conditions of 1 h and 25 °C.

#### 2.1.2. Solubility

Solubility is one of the properties of interest in chemical processes, particularly solubility in water, since aqueous solubility helps minimize the use of organic solvents that contribute to environmental pollution [45,46]. The solubility of commercial chitosan has been reported as a function of the degree of deacetylation of the polymer, as it is related to the number of deacetylated units, going from insoluble chitin (deacetylation < 50%) to a chitosan soluble in acidic aqueous media (pH < 6.5), which is attributed to the protonation of the amino groups [47]. In this research, we sought to determine the influence of the degrees of modification arising from the different synthesis conditions on the solubility of the CTS.

**Figure 4:**
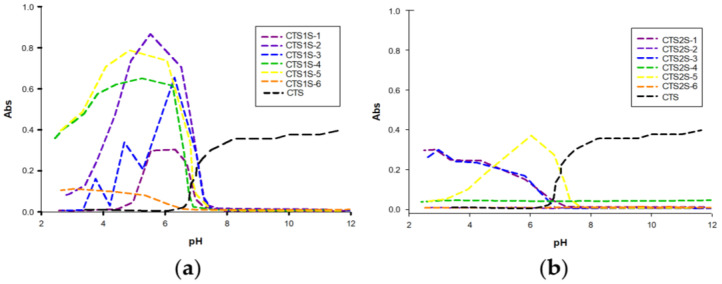
Absorbance results for polymers modified with sulfonic groups. (**a**) Polymers obtained by modifying chitosan with FB1S. (**b**) Polymers obtained by modifying chitosan with FB2S.

The absorbance results obtained as a function of pH are shown in Figure 4, and are complemented by Figure S3, constructed from the transmittance results. In Figure 4, a significant difference can be seen in the absorbance between the two CTS derivatives, with the polymer modified with FB1S (CTS1S) being soluble at basic pH and in most cases insoluble at acidic pH, contrary to CTS, which at pH > 6 is insoluble, due to the high formation of hydrogen bonds and generating curling. For the polymers obtained from the modification with FB2S (CTS2S), solubility over a wide range of pH values is evident, resulting from introduction of the disulfonated aryls.

The solubility behavior can be associated with the presence of sulfonic and amino groups simultaneously, giving the polymer polyampholytic behavior. The solubility at basic pH is justified by the presence of sulfonic groups (pKa = ~2.5), while at a pH below 5.0, the amino groups of chitosan are protonated [18,47]. However, the differences between one group of modifications and the other can be attributed both to the degree of modification and consequently the availability of amino groups that favor solubility at acidic pH, the effect of the π-π interactions of the aryl groups, and the repulsions between the sulfonic groups, which allows the solubility to be preserved in the case of CTS2S in all pH ranges [21].

Overall, it was possible to achieve different degrees of modification to CTS under different conditions of temperature and reaction time. The qualitative and semiquantitative analyses lead to the conclusion that 1 h and 25 °C are the conditions that give rise to polymers with a higher degree of modification, which means lower economic expenses in terms of energy and time, thus favoring a friendlier process.

### 2.2. Preparation of Polyampholytic Hydrogels

**Figure 5:**
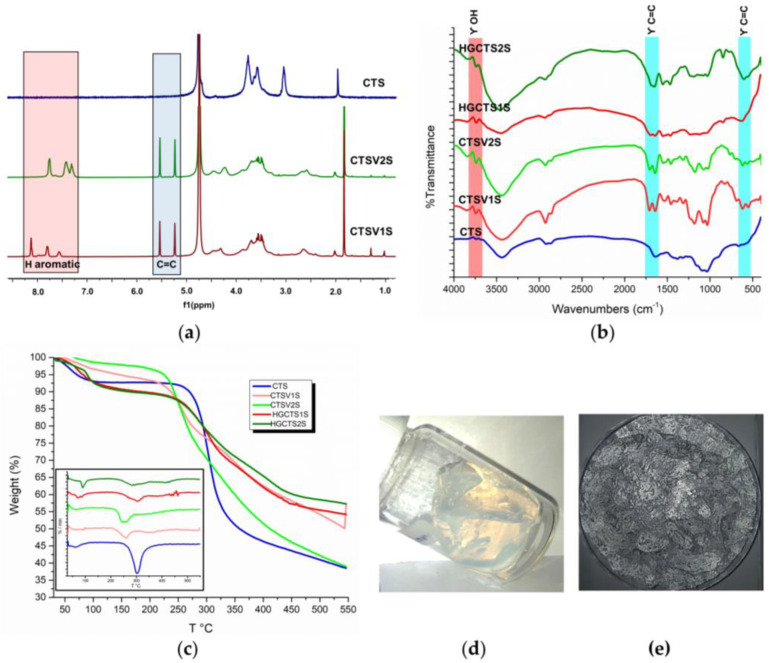
(**a**) ^1^H-NMR spectra of CTS and GMA modified polymers. (**b**) FTIR spectra for CTS, GMA-modified polymer and obtained hydrogels. (**c**) TGA and DTG polymer modified with GMA and hydrogels obtained. (**d**,**e**) Image of the hydrogel at the end of the reaction and after drying, respectively.

Starting from the chitosan modified using the conditions of 1 h and 25 °C, the modification was continued to introduce methacrylate groups for radical polymerization. CTS1S and CTS2S were reacted with GMA at pH 6 and 50 °C for 24 h. The 1H-NMR spectroscopic analysis of the resulting polymers CTSV1S (monosulfonic) and CTSV2S (disulfonic) polymers showed the appearance of new peaks between 5.0 and 5.5 ppm corresponding to the methacrylate groups (Figure 5a).

The NMR analysis agreed with the FTIR analysis (Figure 5b), where three bands of interest were identified, the first two corresponding to the C=C stretching signals between 560 and 630 cm^−1^ and 1325–1396 cm^−1^ [48,49]; and the third, associated with the stretching of OH between 3200 and 3700 cm^−1^. Methacrylation with GMA can result from two different mechanisms, transesterification or opening of the epoxide ring, the latter being the preferred one in acidic media. The reaction results allow us to conclude that the mechanism is the opening of the epoxide rings by the amino groups (still available) and the alkoxy groups. Nucleophilic attack by the amino groups is preferred, with 81 and 66% of the remaining primary amino groups of CTS1S and CTS2S, respectively, functionalized with the GMA. Following treatment with glycidyl methacrylate, 3.39 mmol/g and 2.21 mmol/g of the remaining primary amine groups in the mono- and disulfonic polymers, respectively, were substituted. It evidences an occupation of the GMA by the available amino groups of 3.39 mmol/g of amine for CTS1S and 2.21 mmol/g of amine for CTS2S, which leads to a greater modification of the GMA in the CTS1S.The degree of modification with glycidyl methacrylate (GMA) was calculated using the relationship between the integrals of the NMR signals, and the protons of GMA and CTS [20,49].

**Figure 6:**
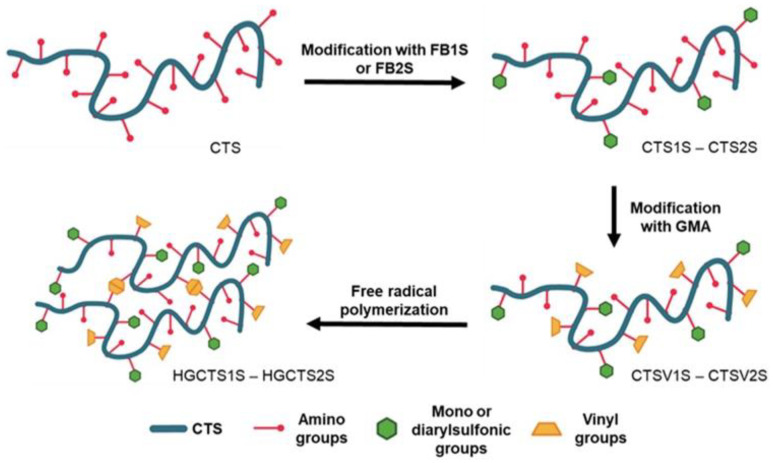
Preparation of HGCTS1S and HGCTS2S. CTS: 4.93, CTS1S: 1.13, CTS2S: 3.35, CTSV1S: 0.79, and CTSV2S: 1.13 mmol/g of primary amine, respectively.

After confirming the modifications with GMA, given the presence of alkene carbon signals, the CTSV1S and CTSV2S were polymerized under free radical conditions to obtain the HGCTS1S and HGCTS2S hydrogels, respectively, (Figure 5d). Figure 5b shows the decrease in the signals associated with the vibrations of the C=C bonds for the HG spectra compared to the spectra of the CTSV1S and CTSV2S polymers, arising from their consumption in the radical polymerization [50]. The process of obtaining the polyampholytic hydrogel is summarized in Figure 6.

The results of the TGA carried out for the sulfonated macromonomers and the hydrogels after several days of drying are shown in Figure 5c. In general, the modified polymers (CTSV1S and CTSV2S) and the hydrogels present similar behaviors to each other with three stages of thermal decomposition and differ from what was observed for the commercial CTS sample, which only shows two stages as mentioned in the previous section. The first stage corresponds to the loss of water, which in the case of the HGs, is larger in magnitude based on the derived thermogravimetry (DTG) (Figure 5c), as a result of the increased hydrophilic capacity of the materials. The second loss for the polymers occurs at 240 and 260 °C, and for the HGs at 305 and 284 °C for HGCTS1S and HGCTS2S, respectively. The differences in behavior for the polymers versus HGs can be attributed to variations in intermolecular interactions. In the case of the polymers, sulfonation can interfere with the formation of hydrogen bonds, whereas in the HGs, these units are now covalently intertwined, favoring thermal stability, which is evident in the residual mass obtained for the HG [50,51].

**Figure 7:**
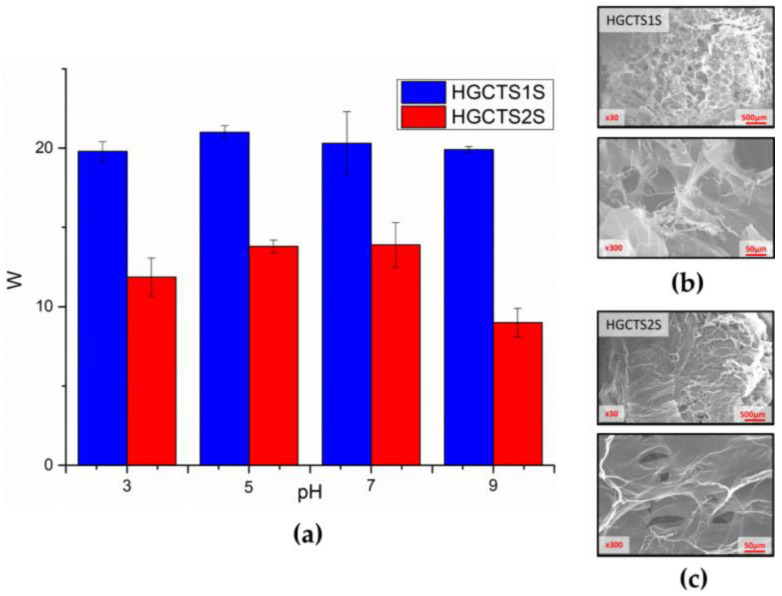
(**a**) Swelling degrees of hydrogels as a function of pH (3, 5, 7, and 9). Images achieved with SEM (30× and 300×) for the (**b**) HGCTS1S and (**c**) HGCTS2S. 2.3. Application in Pollutant Removal.

To evaluate the water absorption capacity, swelling tests for the hydrogels were carried out at four different pH values. Figure 7a shows the degrees of swelling (W) for each hydrogel at different pH values after 24 h, with the HGCTS1S being the one that shows a greater water absorption capacity (20 to 21-fold the initial mass), reaching in almost all cases double those achieved by the HGCTS2S. These results can be associated with both the degree of sulfonate functionalization, which affects the hydrophilicity of the material, and therefore its water absorption capacity, and the degree of cross-linking of the materials. This is related to the degree of modification with GMA, associated with a greater availability of vinyl bonds that participate in the radical addition reaction.

The swelling was also corroborated by images obtained by high-resolution scanning electron microscopy (SEM) (Figure 7b,c), with which the surface morphology of the HG was characterized. Greater roughness was evident in the case of HGCTS1S, associated with a greater degree of modification as has already been reported in other investigations [52,53,54], in this case, corresponding to the addition of benzyl monosulfonate and disulfonate groups to the main column of chitosan. This is consistent with the results obtained from BET and BJH analyses, which reveal significant differences in the porous structure of the HGCTS1S and HGCTS2S hydrogels, despite their synthesis. HGCTS1S has a surface area of 2.88 m^2^/g and an average pore size of 8.79 nm, placing it within the mesoporous range according to the IUPAC classification. In contrast, HGCTS2S displays a very low surface area of only 0.04 m^2^/g and no detectable pore distribution, suggesting a compact or non-porous structure from the perspective of nitrogen adsorption analysis.

Rheological studies of the complex modulus account for the total resistance against the applied deformation (Figure S4), revealing better stability of HGCTS1S, compared to HGCTS2S. This is related to the functionalization of amino groups after modification with GMA, so the availability of vinyl groups that participate in radical polymerization favors cross-linking, reflected in a greater storage modulus (G′). A higher G′ implies enhanced elasticity and mechanical integrity, which are essential for the hydrogel to maintain its three-dimensional network during practical use, especially under repeated compression or handling. Although it is known that greater cross-linking generates a lower water absorption capacity, in this case, the low stability of HGCTS2S could impact an absorption-desorption balance, which reduces the degree of swelling.

Specifically, HGCTS1S, with its higher crosslinking density and elastic modulus, is expected to show better regeneration efficiency due to its ability to retain its structure over multiple cycles. Furthermore, its increased swelling favors interactions such as hydrogen bonding and hydrophobic interactions. In contrast, the denser and stiffer network of HGCTS2S could limit the accessibility of active sites, affecting its adsorption efficiency. Taking into account that both materials were developed under the same conditions, from the methacrylation process to obtaining the hydrogel, the variations in stability and water absorption capacity can be attributed to steric impediments given the presence of more sulfate groups. This is attributed to steric hindrance resulting from the higher number of disulfonate groups, which can restrict the mobility of the polymer network [55,56,57].

**Figure 8:**
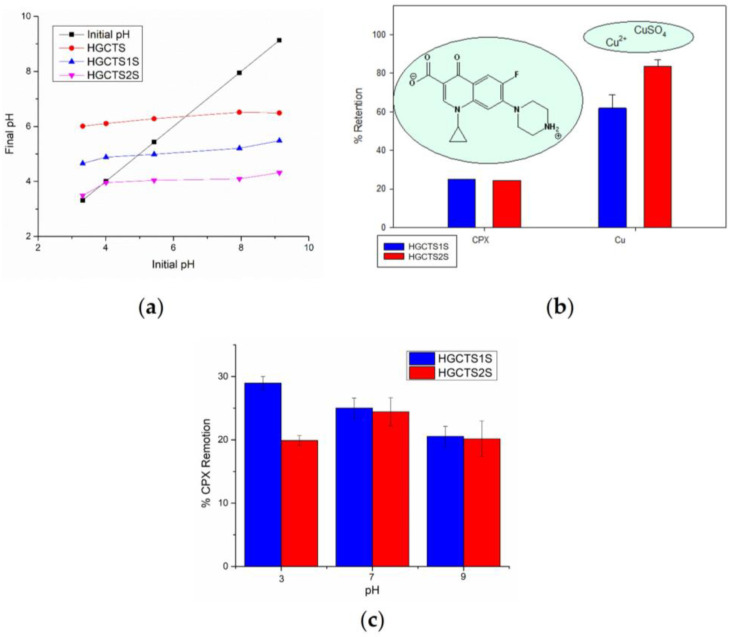
(**a**) Point of zero charge curve of hydrogels. (**b**) Retention percentages and chemical structures of the aqueous contaminants studied. (**c**) Ciprofloxacin removal percentage at different pH values.

A first approach to the possible applications of these materials for the removal of different types of contaminants in aqueous matrices was carried out by performing removal tests on two types of contaminants of interest present in aqueous systems: the antibiotic CPX and Cu^2+^. These tests were carried out at pH 7 in order to emulate natural conditions and based on previous studies where this pH is reported as optimal for the removal of Cu^2+^ and CPX species in aqueous media [58,59,60,61]. The removal percentages achieved after three hours of contact for these contaminants are presented in Figure 8b. The results showed significant differences between CPX versus Cu^2+^, with the adsorption percentages achieved for Cu^2+^ being considerably higher (61.8 ± 1.8–83.7 ± 0.3%) than for CPX (25.0 ± 0.01–24.4 ± 0.01%). To further explore the influence of pH on CPX adsorption and evaluate the applicability of the hydrogels under variable environmental conditions, additional adsorption tests were carried out at pH 3, 7, and 9. The results, shown in Figure 8c, indicate that pH has a moderate but significant effect on CPX removal efficiency. For HGCTS1S, the highest adsorption was observed at pH 3 (28.9 ± 1.06 %), with a slight decrease at pH 7 (25.0 ± 1.59 %), and further reduction at pH 9 (20.5 ± 1.59 %). In contrast, HGCTS2S showed 19.9 ± 0.79 % at pH 3, a maximum at pH 7 (24.4 ± 2.23 %), and 20.1 ± 2.83 % at pH 9.

The slightly better performance of HGCTS1S at this pH may be due to lower steric hindrance compared to HGCTS2S. At pH 7, CPX adopts a zwitterionic form where electrostatic interactions are minimized and other mechanisms, such as hydrogen bonding or hydrophobic interactions, could dominate. At pH 9, the antibiotic becomes negatively charged, which may result in repulsion from the negatively charged hydrogel surfaces, explaining the drop in removal efficiency.

In order to understand the removal mechanism, experiments were carried out for the determination of the zero charge point (pH_ZCP_) for both the chitosan hydrogel (HGCTS) and for each of the sulfonated chitosan-based hydrogels as presented in Figure 8a. The results obtained suggest that the modification with sulfonic groups considerably modifies the pH_ZCP_, being 6.4, 5.0, and 3.9 for HGCTS, HGCTS1S, and HGCTS2S, respectively. Since pH_ZCP_ is the pH at which the value of the net surface charge of the adsorbent is zero, it is understood that below this point, positive charges predominate, and above these, negative charges predominate. The results confirm the significant increase in the arrangement of negative charges on the surface of the material associated with the presence of mono and disulfonic groups, being greater in the case of the latter.

The pH_ZCP_ results correlate with the results of the percentage of removal achieved by each of these materials, because the differences in the retention of the two contaminants by the hydrogels can be attributed to their different chemical structures and properties of each contaminant. On the one hand, CPX has pKa values of 6 and 8.7, with an isoelectric point of ~7.1 so at pH 7 the antibiotic molecule is largely in the zwitterionic state [41,62], so repulsive or neutral electrostatic interactions may dominate. In the case of the copper ion, according to its speciation, at pH 6 it is present mainly as a free Cu^2+^ ion in solution and in a lower percentage as CuSO_4_ [63]. Taking into account the pH_ZCP_, it can be concluded that the density of anionic charges in the material given the modification with sulfonic groups provides the material with anionic characteristics at pH 7, so that copper ions (Cu^2+^) benefit by achieving greater retention. Additionally, the differences between both hydrogels are attributed to the fact that HGCTS2S has a greater availability of anionic groups than HGCTS1S (also evidenced in pH_ZCP_), so the adsorption of contaminants in the cationic state such as Cu^2+^ is favored over that of zwitterionic species such as CPX in which they could be impeded by electrostatic repulsions.

These results allow us to know that the modification of chitosan with sulfonic groups favored not only the solubility and water absorption capacity and therefore its swelling, but also when applied preliminarily for the removal of contaminants, it favors the adsorption of contaminants of a cationic nature such as Cu^2+^. As a result of this research, it is also suggested to continue exploring the applications of this type of materials, evaluating different functionalization relationships to enhance the polyampholytic behavior of the hydrogel, such as evaluating different modifications to obtain higher percentages in terms of the elimination of fluoroquinolone antibiotics.

## 3. Conclusions

This study successfully demonstrated the synthesis of polyampholytic hydrogels from chitosan derivatives functionalized with mono- and disulfonic groups. The hydrogels displayed excellent swelling properties and high adsorption efficiencies for copper ions, making them promising candidates for the removal of heavy metals from wastewater. The best degrees of modification with mono- and disulfonic aryl groups were achieved in 1 h at room temperature, compared to longer time periods or higher temperatures. The hydrogel that demonstrated greater water absorption capability was the hydrogel with aryl-monosulfonate groups, which underwent twice as much swelling as the disulfonic one. This swelling may be associated with differing degrees of functionalization with the sulfonic acids and the cross-linkable methacrylate moieties, as corroborated by ^1^H-NMR spectroscopy, EDS, and TGA studies. For the CPX and Cu^2+^ removal studies, the aryl-disulfonic chitosan hydrogel favored the adsorption of copper with 83% removal compared to 62% in the case of the hydrogel with aryl-monosulfonate groups. For the CPX at pH 7.0, a removal degree of 25% was achieved, with its lower removal efficiency compared to Cu^2+^ attributed to its zwitterionic rather than cationic state. In conclusion, the modification with anionic groups allowed the preparation of polyampholyte hydrogels, which showed that they can be materials with potential use in the processes of eliminating mainly cationic contaminants of organic and inorganic origin in aqueous systems. It is important to note that this study represents an initial approximation of the hydrogel’s potential as an adsorbent for ciprofloxacin and copper ions. Further investigations, including detailed kinetic and isotherm analyses, will be essential to optimize the adsorption performance and fully elucidate the adsorption mechanisms. These studies will be conducted in future work to provide a comprehensive understanding of the hydrogel’s capabilities under various conditions.

## 4. Materials and Methods

### 4.1. General Materials and Procedures

To obtain the zwitterionic polyelectrolyte, chitosan 98% *w*/*w* (CTS) was used with a deacetylation degree of 79.7% (4.93 mmol/g of amino groups) calculated conductometrically, ash percentage of 0.2–0.9%, and molar mass of 150–300 kg/mol, supplied by Quitoquimica (Chile). Sodium cyanoborohydride 95% (NaBH_3_CN), 2-formylbenzylsulfonic acid sodium salt 95% (FB1S), 4-formyl-1,3-benzenedisulfonic acid 97% (FB2S), glycidyl methacrylate (GMA) 97%, and ammonium persulfate (APS) were purchased from Sigma-Aldrich, Chile. Also, 1 M NaOH, 0.1 M HCl, N,N,N′,N′-tetramethyl ethylenediamine (TEMED) 99%, and methanol (99%) were purchased from Merck, Chile. Glacial acetic acid (99.8%) was obtained from Winkler Ltd., Chile.

### 4.2. CTS Characterization: Potentiometric Measurement of Deacetylation Degree (DDA) Determination, and ^13^C-NMR

The polymer (0.2 g) was dissolved under stirring in 5 mL of HCl (1.0 M). Then, 450 mL of 0.001 M NaCl was added and stirred to achieve a uniform solution. This solution was titrated conductometrically with 60 mL of standardized 0.1 M NaOH. The titration was carried out by discharging 0.5 mL of 0.1 M NaOH for each measurement. Measurements were performed by using Thermo Scientific Orion 5-Star Plus (Thermo Scientific, Waltham, WA, USA). The DDA of CTS was calculated using Equation (2) [64,65]. (Figure S1, Equation (2)). The ^13^C NMR spectra were detected by 0.1 M CF_3_COOD as solvents, and the NMR spectra was analyzed by MestReNova software v14.2.3-29241 (Figure S2) [43,66,67].

DDA = (C_NaOH_ × ∆V_NaOH_ × 16)/(m_CTS_ × 0.0994) (2)

DDA = degree of deacetylation
C_NaOH_ = NaOH concentration (M)
∆V_NaOH_ = volume difference between the two inflection points (L)
m_CTS_ = mass of the analyzed chitosan (g)

### 4.3. Functionalization of CTS with Sodium Sulfonates

**Figure 9:**
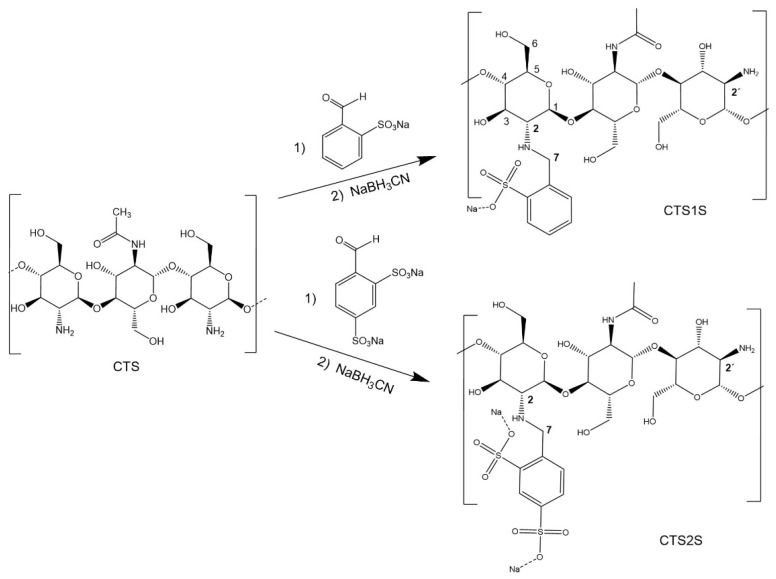
Synthesis of benzyl monosulfonate and disulfonate derivatives CTS1S and CTS2S.

The modification of chitosan (CTS) with sulfonic groups (Figure 9) was carried out by reductive amination [24,48]; varying the time (1.0, 8.0 and 24.0 h) and temperature (25 and 60 °C) at a fixed molar ratio of 1.5 equivalents of sulfonic acid/available amino group, 100 and 80 mg of CTS were dissolved in 10 mL of a 1% (*v*/*v*) aqueous acetic acid solution and subsequently 9 mL of methanol were added for modification with FB1S and FB2S, respectively. After obtaining a homogeneous mixture, FB1S (0.74 mmol) and FB2S (0.59 mmol), dissolved in 1 mL of water, were added to separate flasks. The reaction mixture was stirred for 3 min and then finally 60 mg of NaBH_3_CN are added, followed by stirring for 1, 8 or 24 h, at temperatures of 25 or 60 °C. The product was then dialyzed for four days using a cellulose membrane regenerated (Sigma-Aldrich, St. Louis, MI, USA) with a molecular weight cut-off of 12–14 kDa. Finally, the modified polymers were frozen and then lyophilized for 24 h.

Table 2 shows the codes that refer to the different conditions explored.

The modified CTSs obtained were characterized by ^1^H-NMR spectroscopy, dissolving 10 mg of polymer in 600 μL of 0.01 M NaOD. Through EDS analysis, the percentage composition of carbon, oxygen, nitrogen, and sulfur was determined by scanning electron microscopy (JEOL JSM 6010 Plus/LV SEM, JEOL Ltd., Tokyo, Japan) after the samples were coated in gold with LUXOR Gold Coater equipment. Thermogravimetric analysis (TGA) was carried out using a Netzsch TG 209F1 iris 220-12-0045-L instrument (NETZSCH-Gerätebau GmbH, Selb, Germany) with nitrogen flow, in a range of 25 to 550 °C, with a heating rate of 10 °C min^−1^. Fourier transform infrared (FTIR) spectroscopy was performed using a Perkin Elmer 1760-X spectrophotometer (PerkinElmer, Inc., Waltham, MA, USA) with a range 4000–400 cm^−1^ and using a KBr pellet of the material.

To determine the solubility as a function of pH, a turbidimetric technique with an ultraviolet-visible (UV-Vis) spectrophotometer (UVmini-1240, Shimadzu, Tokyo, Japan) operating at a wavelength of 660 nm was carried out. Solutions (2 mg·mL^−1^) of the chitosan sulfonate derivatives were prepared in 0.01 mol L^−1^ NaOH, and then the pH was adjusted from 11.0 to 3.0 with the addition of 0.05 mol L^−1^ HCl.

### 4.4. Preparation of Polyampholytic Hydrogels

Preparation of the hydrogels was carried out in two stages: (*i)* synthesis of the methacrylate-functionalized macromonomer modified with sulfonic groups and (*ii)* preparation of the hydrogels via free radical polymerization. To synthesize the macromonomer, 0.5 g of either the chitosan with mono (CTS1S) or disulfonic (CTS2S) groups was dissolved in 50 mL of water and the pH was adjusted to 6 using HCl (0.1M). The reaction was heated to reflux at 50 °C and then 1.5 mL of glycidyl methacrylate (GMA) was added slowly dropwise under vigorous stirring until a homogeneous solution was obtained. Stirring (200 rpm) was maintained for 24 h [50,68], and then the polymer was purified by dialysis, using a regenerated cellulose membrane with a molecular weight cut-off of 12–14 kDa for 3 days with water exchange every ~4 h. Then, the sample was frozen and lyophilized. The products were characterized by TGA, FTIR spectroscopy, and ^1^H-NMR spectroscopy.

The hydrogels were prepared by dissolving 1.0 g of the mono- or disulfonic macromonomer (CTSV1S or CTSV2S, respectively) in 25 mL of 1 M NaOH in a Schlenk tube. This solution was stirred until a homogeneous mixture was obtained, and then 200 mg of the N,N-methylenebisacrylamide (BIS) cross-linker was added while stirring vigorously. Once homogenized, nitrogen was bubbled through the solution for 10 min and then 100 mg of ammonium persulfate (APS) as an initiator was added with constant stirring, followed by 100 mg of TEMED as a catalyst. The solution was heated at 50 °C resulting in gelation after ~30 min and then the heating was continued for 4 h to allow all the reagents to [50]. The resulting gel was washed extensively with water until a neutral pH was achieved, and then it was dried at 30 °C for 24 h, and finally pulverized and sieved, to produce particles with diameters between 100 and 280 μm.

### 4.5. Characterization of Polyampholytic Hydrogels

The materials obtained were characterized by TGA and FTIR. Additionally, the morphologies were observed by scanning electron microscopy (SEM-mapping; JEOL JSM 6010 Plus/LV SEM, JEOL Ltd., Tokyo, Japan), freezing and lyophilizing the fully swollen hydrogels, to subsequently coat them in gold using the low-vacuum sputter coating technique. Finally, swelling tests were carried out in water at 4 different pH values (3.0, 5.0, 7.0, and 9.0), weighing dry hydrogel pieces and placing them in water, to determine swelling percentages after 24 h (Equation (3)) [69,70]. The swelling was then calculated using Equation (3).

W = (W_t_ − W_0_)/W_0_ (3)

W = Degree of swelling
W_0_ = Dry weight
W_t_ = Weight over time

Additionally, rheological tests were carried out using parallel plates (DHR-3/TA instruments rheometer, TA Instruments, New Castle, DE, USA) ETC/20 mm standard, using oscillatory scanning measurements at a temperature of 25 °C, frequency of 1 Hz and deformation of 0.001 to 500% in triplicate to determine the Storage modulus, Loss modulus, Complex viscosity, and Complex modulus.

Finally, the pH of the zero charge point (pH_ZCP_) of the hydrogels obtained was determined. For this, 0.1 M NaCl solutions with pH between 3 and 9 (adjusted with HCl or NaOH) were prepared. Then, 10 mL of these solutions were taken, and 0.08 g of each hydrogel was added. The suspensions obtained were stirred for 24 h. Then, the dispersions were filtered and the final pH of the solutions was determined. The pH_ZCP_ value of the hydrogels was found from the intersection of the final pH vs. initial pH curve with the abscissa.

### 4.6. Application of the Hydrogels to Adsorption of Ciprofloxacin and Copper Ions in Aqueous Systems

The application of the polyampholytic hydrogels for the adsorption of contaminants in aqueous systems was carried out at pH 7 from a 20 mg L^−1^ solution of each contaminant: ciprofloxacin (CPX) and Cu^2+^ (from the salt of copper sulfate pentahydrate). The removal calculations for Cu^2+^ were carried out from the Cu^2+^ in solution. A total of 20 mg of hydrogel in triplicate was placed in contact with 10 mL of each solution for 3 h under constant stirring at a temperature of 25 °C. To monitor both contaminants, the respective calibration curves were constructed that varied from 1 to 20 ppm. CPX monitoring was carried out by UV spectroscopy at 271 nm using an Evolution OnePlus UV-Vis Spectophotometer (Thermo Scientific, Waltham, MA, USA), while for Cu^2+^ atomic absorption spectroscopy was performed using a PinAAcle 900F instrument (Perkin Elmer, Waltham, MA, USA) monitoring the removal as a function of the coverage concentration in solution.

## Figures and Tables

**Table 1:** Results obtained by ^1^H-NMR spectroscopy, SEM-EDS, and TGA for biopolymers modified with mono- and disulfonic groups under different time and temperature conditions.

	Samples	1 h—25 °C	1 h—60 °C	8 h—25 °C	8 h—60 °C	24 h—25 °C	24 h—60 °C
^1^H-NMR	%DS ^a^ CTS1S	15	11	13	11	11	8
	%DS ^a^ CTS2S	32	21	26	22	39	16
SEM-EDS	%S ^b^ CTS1S	12.29	9.76	2.77	8.73	4.74	11.11
	%S ^b^ CTS2S	11.15	13.13	7.86	10.98	12.21	14.83
TGA	%Wt ^c^ CTS1S	21	20	18	13	16	17
	%Wt ^c^ CTS2S	22	11	13	19	14	16

^a^ Degree of substitution. ^b^ Percentage of sulfur. ^c^ Percent of weight loss of mono or disulfonic aryl.

**Table 2:** Codes referring to the different time and temperature conditions used to prepare polymers modified with mono- and disulfonic groups.

	1 h 25 °C	1 h 60 °C	8 h 25 °C	8 h 60 °C	24 h 25 °C	24 h 60 °C
FB1S	CTS1S-1	CTS1S-2	CTS1S-3	CTS1S-4	CTS1S-5	CTS1S-6
FB2S	CTS2S-1	CTS2S-2	CTS2S-3	CTS2S-4	CTS2S-5	CTS2S-6

## Data Availability

The data presented in this study are available on request from the corresponding author.

## Supplementary Materials

The following supporting information can be downloaded at: https://www.mdpi.com/article/10.3390/gels11080622/s1, Figure S1. Conductivity for the determination of the degree of deacetylation (DDA). Figure S2. 13C-NMR spectra of chitosan samples. Figure S3. Transmittance percentage results for polymers modified with sulfonic groups, (a) Polymers obtained by modifying chitosan with FB1S. (b) Polymers obtained by modifying chitosan with FB2S. Figure S4. Rheological characterization, (a) complex modulus by Oscillation strain from 0.001% to 500% (b) Comparison of the complex modulus at 0.01% of Oscillation strain.

## Abbreviations

The following abbreviations are used in this manuscript:

GMA	Glycidyl methacrylate
CTS	Chitosan
BIS	N,N-methylenebisacrylamide
APS	Ammonium persulfate
CPX	Ciprofloxacin
HG	Hydrogel
FB1S	2-formylbenzylsulfonic acid sodium salt
FB2S	4-formyl-1,3-benzenedisulfonic acid
TEMED	N,N,N’,N’-tetramethyl ethylenediamine
pH_ZCP_	pH of zero charge point
DDA	Degree of deacetylation of the starting chitosan
DS	Degree of substitution
CTS1S	Chitosan modified with FB1S
CTS2S	Chitosan modified with FB2S
CTSV	Chitosan modified with GMA
CTSV1S	CTS1S modified with GMA
CTSV2S	CTS2S modified with GMA
HGCTS	Hydrogel obtained from CTS
HGCTS1S	Hydrogel obtained from CTSV1S
HGCTS2S	Hydrogel obtained from CTSV2S
^1^H-NMR	Proton nuclear magnetic resonance
FTIR	Fourier transform infrared
SEM	Scanning electron microscopy
TGA	Thermogravimetric analysis
