# Supplementary material for: Polyampholytic Hydrogels from Chitosan Macromonomers with Aryl-Mono and Di-Sulfonated Groups: An Approach to the Removal of Copper Ions and Ciprofloxacin in Aqueous Solutions
Source: Gels. 2025 Aug 8;11(8):622. doi: 10.3390/gels11080622 (PMC12386152; doi:10.3390/gels11080622)
Supplement: Supplementary file 1 [file gels-11-00622-s001.zip › gels-3571321-supplementary.pdf]

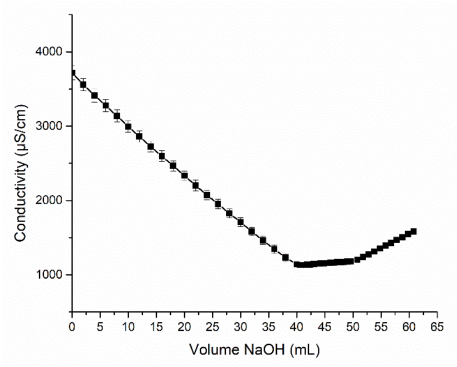

Figure S1. Conductivity for the determination of the degree of deacetylation (DDA).

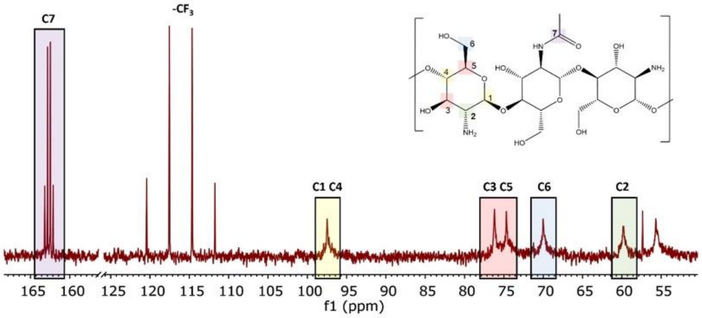

Figure S2. ^{13}C-NMR spectra of chitosan samples.

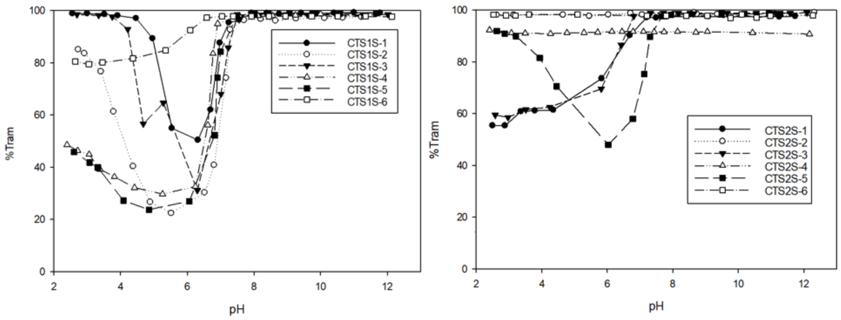

(a) (b)

Figure S3. Transmittance percentage results for polymers modified with sulfonic groups, (a) Polymers obtained by modifying chitosan with FB1S. (b) Polymers obtained by modifying chitosan with FB2S.

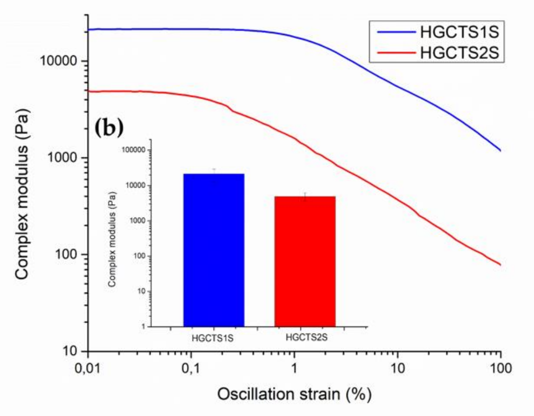

(a)

Figure S4. Rheological characterization, (a) complex modulus by Oscillation strain from 0.001% to 500% (b) Comparison of the complex modulus at 0.01% of Oscillation strain.
